# Automated Analyses of Innate Olfactory Behaviors in Rodents

**DOI:** 10.1371/journal.pone.0093468

**Published:** 2014-04-03

**Authors:** Qiang Qiu, Aaron Scott, Hayley Scheerer, Nirjal Sapkota, Daniel K. Lee, Limei Ma, C. Ron Yu

**Affiliations:** 1 Stowers Institute for Medical Research, Kansas City, Missouri, United States of America; 2 Department of Anatomy and Cell Biology, University of Kansas Medical Center, Kansas City, Kansas, United States of America; Université Lyon, France

## Abstract

Olfaction based behavioral experiments are important for the investigation of sensory coding, perception, decision making and memory formation. The predominant experimental paradigms employ forced choice operant assays, which require associative learning and reinforced training. Animal performance in these assays not only reflects odor perception but also the confidence in decision making and memory. In this study, we describe a versatile and automated setup, “Poking-Registered Olfactory Behavior Evaluation System” (PROBES), which can be adapted to perform multiple olfactory assays. In addition to forced choice assays, we employ this system to examine animal’s innate ability for odor detection, discrimination and preference without elaborate training procedures. These assays provide quantitative measurements of odor discrimination and robust readouts of odor preference. Using PROBES, we find odor detection thresholds are at lower concentrations in naïve animals than those determined by forced choice assays. PROBES-based automated assays provide an efficient way of analyzing innate odor-triggered behaviors.

## Introduction

Behavioral assays are an important component in the study of sensory biology. Experimental paradigms have been developed to study odor detection, discrimination and preference, as well as higher cognitive functions such as decision making and associative learning [1]–[4]. However, designs of olfaction-based experimental paradigms vary significantly in equipment, software and the level of sophistication [5]–[23]. For example, odor-mediated associative learning has been examined with paradigms ranging from simple food digging experiments [13] to assays using computer-controlled pneumatic apparatuses with precise control of odor delivery [8]–[12], [16], [18]–[22].

Learning-based forced choice olfactory tasks, such as Go/No Go and two choice assays, have been widely used to examine odor detection [7], [16], [21], [24], [25], discrimination [8], [26], and decision making. These operant tests require extensive training and cannot be used to probe odor detection and perception in animals that have not been previously exposed to the test odors.

A few methods, such as Y- or T-maze [27]–[30] and cross habituation assays [15], have been developed to evaluate the innate ability of animals to detect and discriminate odors. In these assays, investigation of the odor sources are videotaped and scored manually. There is no automated assay available to reduce factors influencing the experimental outcome, such as human presence and subjective calls in video analysis. Moreover, the employment of multi-chamber apparatuses could also influence the robustness of behavioral assays. Preference evaluation using three-chamber, Y- or T-mazes depends on the assumption that animal will spend equal amounts of time in the two test chambers (or maze arms). This assumption is often not met because terrestrial animals mark territories and have an innate preference for the marked territory [30].

Here, we present an automated system employing olfactometers and a single-chamber design to analyze innate olfactory behaviors. The integrated system, “Poking-Registered Olfactory Behavior Evaluation System” (PROBES), can be used for a variety of olfaction-related experiments. The single behavioral chamber with interchangeable panels can be adapted for different assays, and an integrative software package controls all aspects of the experiments. This system can be used to automate various assays and to provide robust tests of odor detection, discrimination and preference without extensive training. Taking advantage of the new design, we explore the use of this system in a dis-habituation assay [15], [28] to examine odor detection threshold, which is normally evaluated using Go/No Go assay. This new paradigm does not require training, therefore eliminating influence by decision confidence and the valence of the expected reward or punishment [3]. Similarly, we also demonstrate the use of this system in automated evaluation of innate odor preferences.

## Materials and Methods

### Animals

C57BL/6 mice 2–6 months of age were used in the experiments. Animals were maintained on 12∶12 shifted light cycle. For reinforced training, animals were maintained on a water restriction regimen with 1.5–2 ml of drinking water per day for one week before training. All training and tests were performed during the dark cycle. Experimental protocols were approved by the Institutional Animal Care and Use Committee at Stowers Institute and in compliance with the NIH Guide for Care and Use of Animals.

### Odors

Odorants were purchased from Sigma Aldrich at highest purity or at purity >99%. Each odorant was diluted in mineral oil (MO) and placed in odor vials or loaded onto syringe-top filters. Odorants used in this study include: amyl acetate (AA), 2-hexanone (HXO), hexanal (HXH), heptanal (HPH), methyl valerate (MVE), isoamylamine (IAMM), 2-methylbutyric acid (2-MBA), methyl caproate (MCE), *S*-(+)-Carvone (+Car), *R*-(−)-Carvone (−Car) and mouse urine freshly collected on the day of experiments.

### PROBES Olfactometer Construction

Hardware design files, parts list, software source code, compiled program and instruction manuals can be found at the Stowers Institute FTP site: ftp://ftp.stowers.org/pub/yu_lab/PROBES/.

The basic olfactometer design is based on that of Uchida and Mainen [20]. Briefly, each olfactometer consists of two odor banks to allow binary mixture of odors at different ratios. A pressurized clean air stream is split into four lines: a carrier line, one fixed speed line, and two variable speed odor lines. The carrier line is controlled by a mass flow controller (GFCS-012161 from Aalborg; 0–1000 ml/min flow rate) and the odor lines are controlled by three independent mass flow controllers (GFCS-010312 from Aalborg; 0–100 ml/min). The fixed speed line is set at 100 ml/min and can be diverted into one of the two odor banks to provide the highest speed of odor flow. Each of the variable speed (1–99 ml/min) odor lines controls air flow from one odor bank. The maximal combined flow rate from the two banks is 198 ml/min. Each odor bank consists of four independent valves that control air flow through one of four odor vials. The air stream that carries the odor then feeds into a manifold where the main carrier stream flows and, thereby, dilutes the odor according to flow rates. Air flow is controlled by Micro Insert Valves (MIV, Lee Company). Two-way manifold mounted MIVs (LFVA1250310H) are used to control air flow to odor vials. Two-way MIVs (LFVA1230313H) are used to control air flow to each manifold, and three-way MIVs (LFRA1230310H) are used to control the air flow from the flow controller to two-way MIVs.

### Computer Interface

In the latest implementation, one DAQ 6229 and two DAQ 6722 boards (National Instruments) are used to control four olfactometer circuit boards. Printed circuit boards (PCBs) were designed using PCB Artist (1.5.1) and manufactured by Advanced Circuits. PCBs are used to control individual valves, odor flow rates, TTL outputs and receive signals from infrared sensors in the behavior boxes.

### PROBES Software Design

The software package that controls the olfactometers and behavioral apparatuses was developed in LabVIEW 8 professional development environment (National Instrument). The source code was compiled into an executable file (v1.0.5.4) and can be installed on any computer running the Windows XP/7 operating system with LabVIEW runtime engine 8.0.1 (http://www.ni.com/download/labview-run-time-engine-8.0.1/741/en/). Default hardware connections and settings are stored in task and configuration files. These files are included in the installation package and can be edited to accommodate different experimental designs. With this software, up to twelve devices (e.g., behavior boxes) can be controlled at once by a single computer to allow the simultaneous control and recording of multiple experiments.

The software package allows three levels of operations. At the entry level, the end users use the software’s graphic user interface and choose a package to set up and manage the experimental paradigm. The packages contain parameters that determine the logic flow between individual experimental steps and control hardware devices. All experimental paradigms can be saved and reloaded. At the intermediate level, advanced users can change hardware settings to create more elaborate behavioral tasks. Finally, at the source code level, software engineers can make fundamental changes to the software to accommodate more complex tasks, improve memory usage and optimize the software.

For general use, the most basic functions are valve control and conditional valve control. Valve control commands control a set of MIVs that divert the air flow through the pneumatic system to release odors from user-designated odor sources. Users also set the timing and duration of odor release. A built-in function sets the overall flow rate to remain constant. If a total flow rate is set at 1000 ml/min, when one odor port is set to open and deliver a flow of 100 ml/min, the flow speed for the carrier stream will be reduced to 900 ml/min during odor delivery. The carrier stream is reverted back to 1000 ml/min once odor delivery is finished. This setting minimizes any mechanical artifact associated with many odor delivery systems.

Conditional valve control is a set of commands dependent on other inputs. The most common is IR-beam breaking triggered valve actions. Beam-breaking events are registered by analog input channels. Crossing a user determined threshold triggers the opening of designated odor or water valves with user specified delay and duration.

In addition to these two basic functions, several other functions make the software versatile. These include a split function, TTL input and output, conditional splits, and a loop function. The software also includes functions that allow the cloning of a procedure, and a randomized odor port choice function.

### Photo-ionization Detector (PID) Measurement

Odor concentrations were measured by miniPID (200B, Aurora Scientific Inc.). Signals from the miniPID were digitized by MiniDigi-1B (Molecular Devices) at 1 kHz and low pass filtered (10 Hz) in pCLAMP10.3 (Molecular Devices).

### Video Recording and Tracking

Animal behaviors were video recorded through internet infrared cameras (Vivotek Inc) with 640×480 resolution at 30 Hz frame rate. All recordings were performed under infrared illumination. Nose poking activities were visually assessed and manually scored in Observer 7.0 software (Noldus). For the 3-chamber assay, the arena was an 8 cm (w)×60 cm (L)×8 cm (H) chamber divided into three chambers. The two side chambers measured 25 cm long and the middle chamber was10 cm long. Odor vials were placed at the end of the side chambers. Mice were habituated to the behavior chamber for 30 minutes with empty vials prior to the introduction of odor vials. Videos were resampled (15 Hz) and analyzed offline with EthoVision 3.1 (Noldus). Time spent in the each zone during odor presentation (5 or 10 minutes) was calculated.

### Data Analysis

The event log created by the PROBES software package was converted and analyzed using custom-written scripts in MATLAB (MathWorks). ANOVA and pairwise student *t*-test were performed for statistical analysis in OriginPro8.6 (OriginLab).

For threshold detection, the performances of animals at different odor concentrations (*x*) were fitted in MATLAB with NonlinearLeastSquares method using Weibull psychometric function:
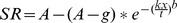
where



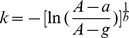



*A* is the maximum performance, *b* is the steepness of the function, and *a* is the threshold concentration. The parameter *g* is the false alarm rate. In Go/No Go tests, *g* is set at 50% as chance level. For habituation/dis-habituation assay g is set at 0%.

## Results

### General Design of PROBES

**Figure 1:**
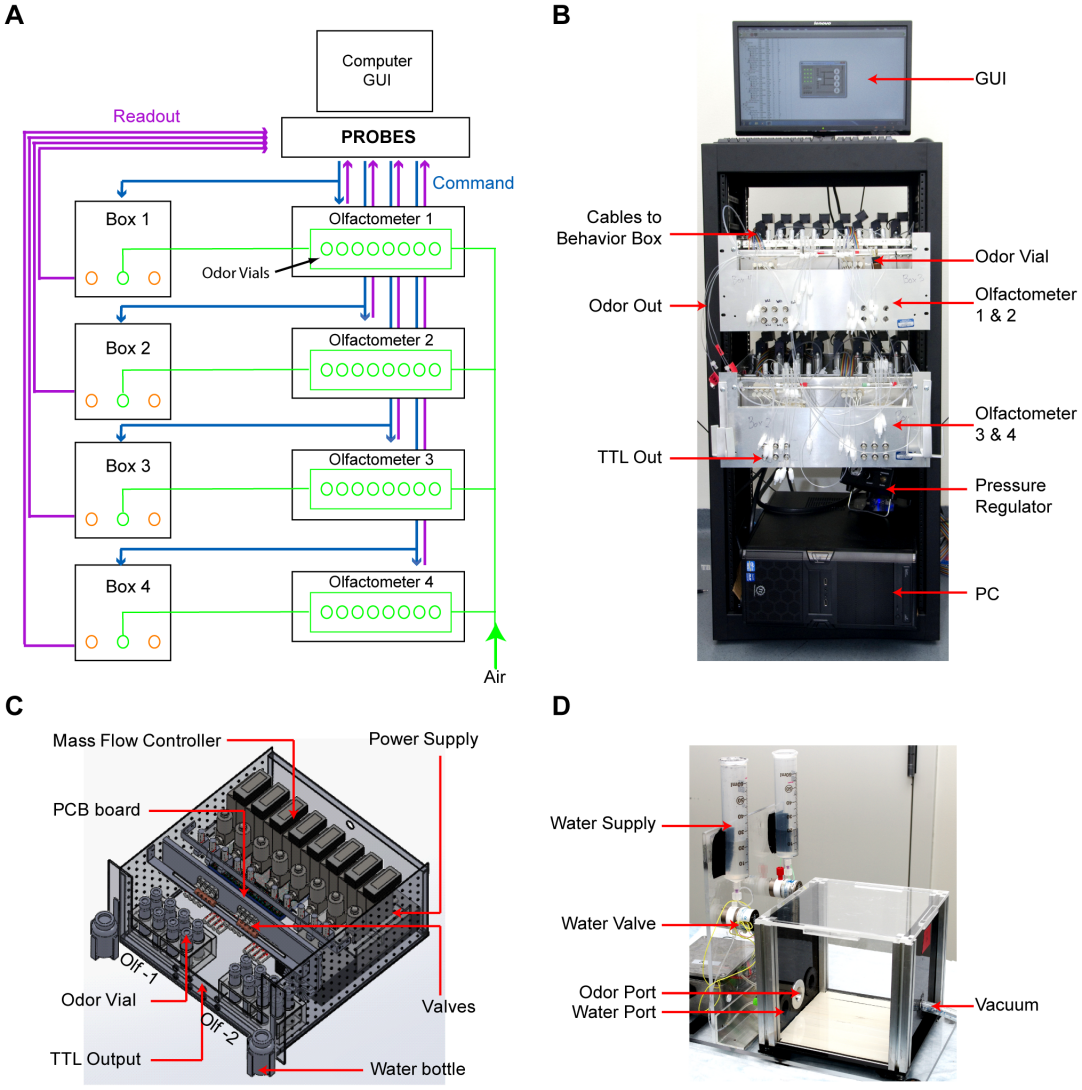
The Design of PROBES. **A**) Schematic illustration of the system. A single PROBES software interface gives commands and integrates readout from the olfactometers and behaviors boxes. Four separate olfactometers and behavioral boxes are independently controlled, and each olfactometer controls eight odor channels. Odor from each olfactometer is delivered to the corresponding behavior box. Readout is indicated by purple lines, commands are indicated by dark blue lines and air flow paths are indicated by green lines. **B**) Photograph of PROBES setup with four olfactometers. Each box contains two independent olfactometers. **C**) Detailed layout of a box containing two separate olfactometers. **D**) A typical behavior box with the triple port configuration for reinforced two-choice assays. Odors are delivered through the middle nose cone while the water reward ports are controlled by two water valves.

The general design of PROBES includes a computer control unit, olfactometers and behavioral boxes (Figure 1A). The apparatus shown in Figure 1B consists of a set of four olfactometers, each connected to a behavioral chamber such that four individual animals can be trained and tested simultaneously (Figure 1B&C). Readout from the behavioral boxes is sent to the computer interface (Figure 1A). Commands and box readouts are saved in a log file.

**Figure 2:**
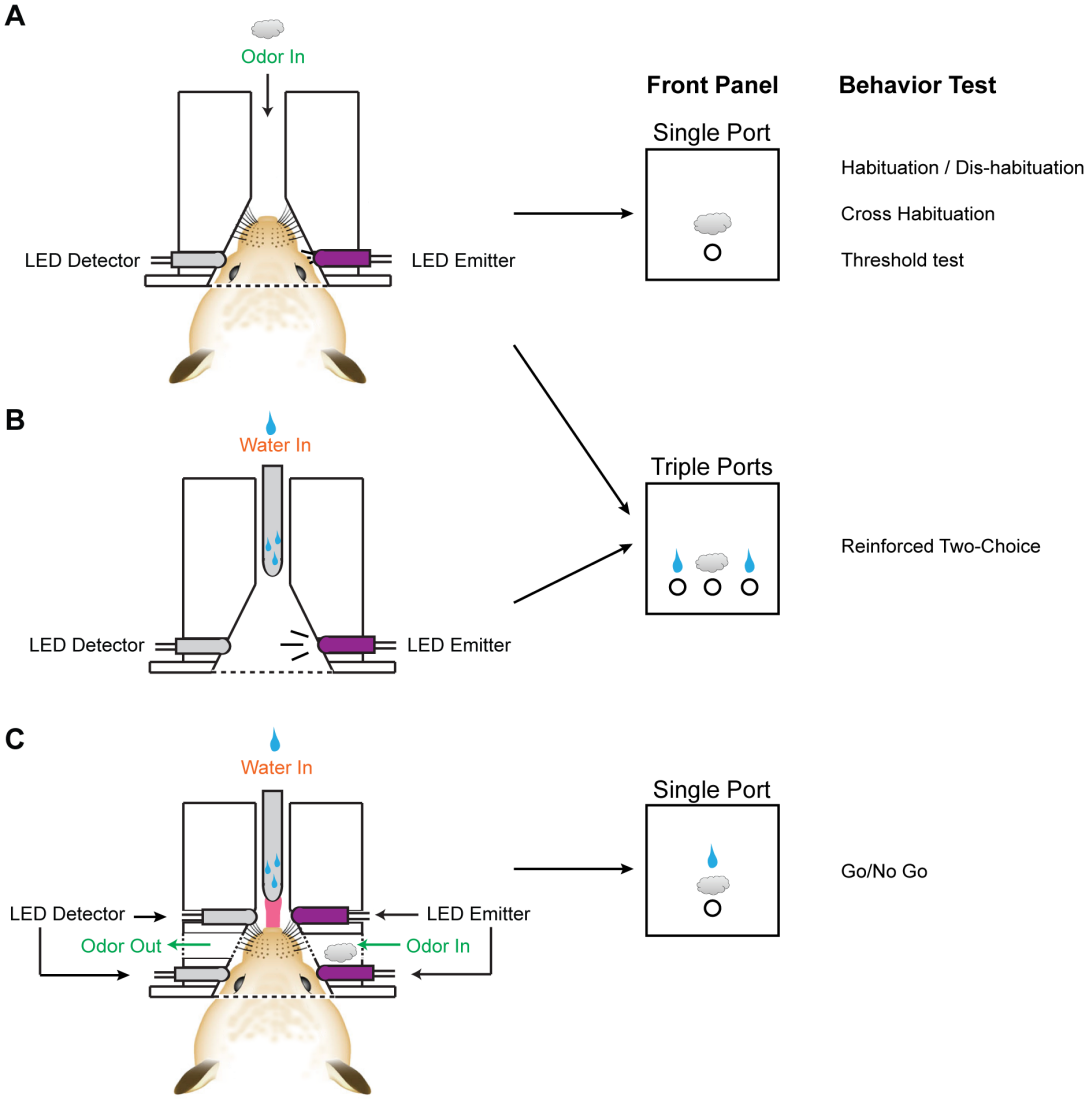
Behavior Box Design. **A**) Single port design with one pair IR LED emitter and detector that can be used to trigger odor delivery. **B**) A triple port design that combines the odor port in **A** with two water ports. A water spout is included in each water port. **C**) A single port design that combines water and odor delivery. Two sets of paired IR LED emitter and detector are implemented to detect nose poking and licking activity.

The behavioral chamber is a simple 20 cm×20 cm×20 cm acrylic box with a interchangeable front panel that can house up to three ports for different behavioral tasks (Figures 1D & 2). The back panel contains a vacuum port to facilitate air exchange inside the chamber. We implemented three configurations to accommodate different behavioral tasks. The first one, shown in Figure 2A, utilizes a port located in the center with a single paired infrared light emitting diode (IR-LED) and IR detector installed. Beam-breaking events indicate nose poking by the animal (Figure 2A). This simple implementation can be used for odor threshold detection, cross habituation and odor preference evaluation.

The second configuration contains three ports with a single central odor port flanked by two water ports (Figure 2B). The software allows water reward to be delivered each time when an animal correctly identifies an odor and pokes the appropriate water port. This configuration is used for reinforced two-choice assay.

In the third configuration, a single port combines both odor and water delivery (Figure 2C). Odor is delivered through a side channel and a water spout is positioned deep in the port. In addition to detecting nose poking events, a second paired IR-emitter and detector is positioned just in front of the water spout to register licking events. The narrow passage in front of the water spout ensures only licking interrupts the IR beam. This configuration allows us to perform the Go/No Go test, in which water-deprived animals can be trained to recognize an odor paired with water reward. Incorrect odor identification results in either no reward or punishment in the form of a mild electric foot-shock.

Thus, by configuring PROBES, we are able to quickly switch among different experimental paradigms. During each experiment, once the animals are placed in the behavioral boxes, no human interaction is needed. All odor delivery, determination of reward/punishment, and registration of different events are automatically controlled by the software. Animals are confined within a single, undivided chamber, reducing spatial bias. Moreover, the automated process allows the user to run up to four separate experiments simultaneously, significantly improving the efficiency of data acquisition.

### Odor Delivery by the Olfactometer

The ability to deliver odors at the desired concentrations and at the precise time is essential for studying behavioral response, sensory physiology and central processing of olfactory information in the brain. Unlike light and sound, which can be controlled precisely from the source, odors are chemicals that diffuse in the air. Many factors along the delivery path influence the odor concentration present at the site of detection.

In our design, odors are diluted in an inert solvent, such as mineral oil. Highly polar organic compounds are diluted in water. During odorant preparation, we fill a 22 ml vial with 5 ml of diluted odor such that each vial has 17 ml headspace, in which odorant equilibrates with air. Air flow through the inlet of the vial pushes odor vapor through the outlet into the carrier air stream.

**Figure 3:**
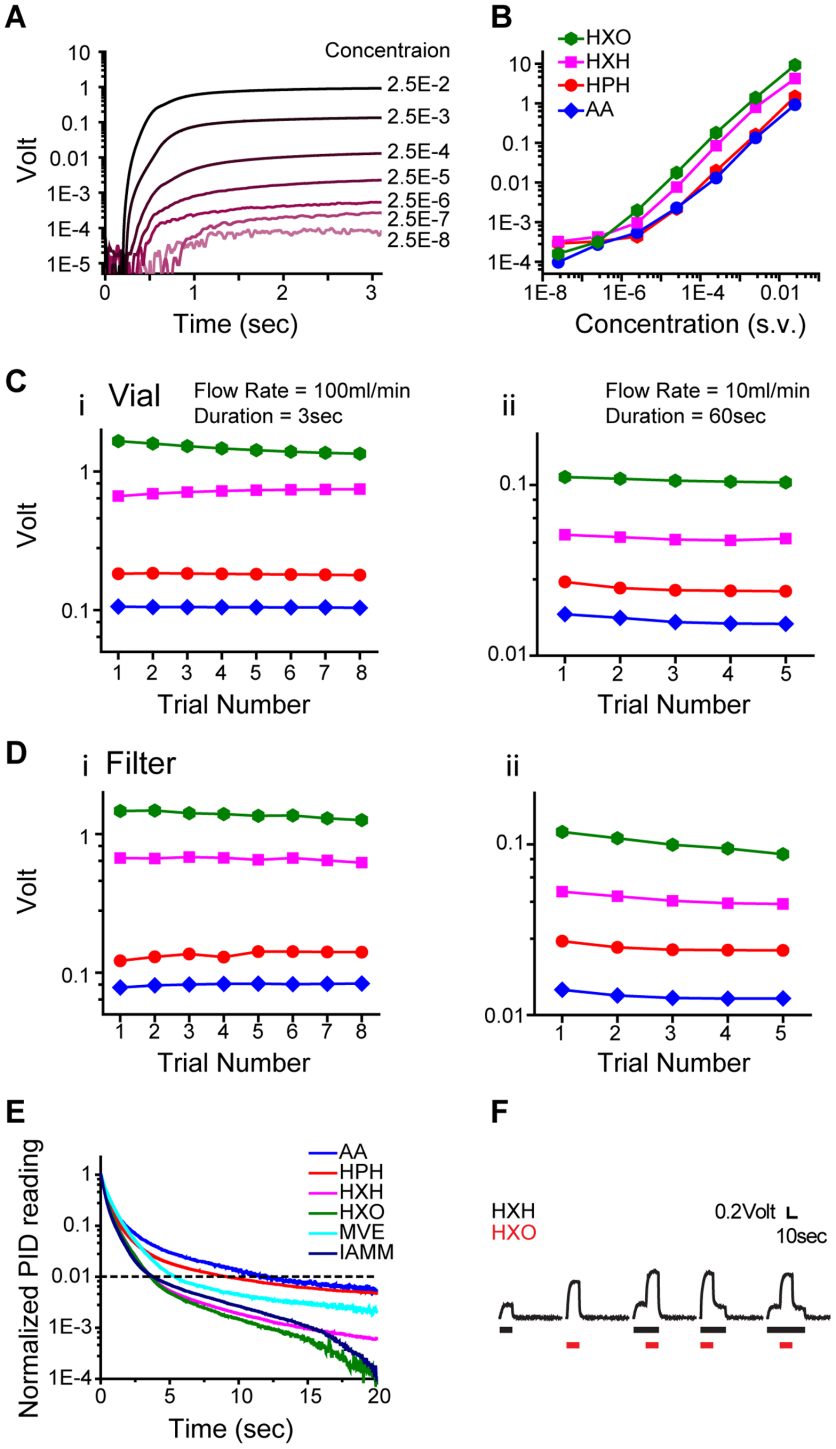
Odor Delivery Controlled by the Olfactometer. **A**) Sample traces of PID measurement of AA at various concentrations. **B**) Dose response curve of four different odors at seven concentrations. **C&D**) PID measurements of repeated odor applications over multiple sessions using single odor vials (**C**) and syringe top filters (**D**). Flow speed and application duration are as indicated. **E**) Normalized PID measurement values as a function of time after odor delivery. Odor concentration: 2.5×10^−3^ s.v. **F**) Sample PID traces of odor mixture delivery using olfactometer. The black bars indicate the delivery of HXH. The red bars indicate the delivery of HXO.

First, we tested the delivery of odor at various concentrations via the olfactometer. We diluted odorants at ratios ranging from 1∶10 to 1∶10^7^ (v/v) in mineral oil. The odor air stream was set at 100 ml/min, and the total air flow was set at 400 ml/min, with the final dilutions of odor in the air ranging from 2.5×10^−2^ to 2.5×10^−8^ saturated vapor (s.v.). PID reading showed a rapid rise in the signal regardless of the odor concentration being tested (Figure 3A). Response amplitude to an odor depended on its volatility and photo-ionization properties. For the four odors tested, we observed that the response amplitudes were proportional to the concentration over a wide range of dilutions (Figure 3A&B). But, at low concentrations, outputs appeared to have reached detection threshold and became noisy and non-linear.

Our olfactometer was designed to perform multiple odor applications during an experiment without the need to change odor vials. Next, we examined whether repeated odor applications would result in rapid odor depletion. The PID reading indicated that odor output remained consistent across multiple, consecutive sessions (Figure 3C). We also used 100 μl odorant solutions applied to syringe top filters as alternative odor sources. Tests conducted using the filter yielded results nearly identical to those using the odor vials (Figure 3D). Additionally, the PID readouts quickly dropped after odor valves were closed (Figure 3E). Within 10 seconds, the reading dropped below 1% of the initial value. The more volatile odors, such as isoamylamine (IAMM, vapor pressure = 51.1 mm/Hg) and HXO (vapor pressure = 12 mmHg), declined faster than the less volatile odors, such as amyl acetate (AA, vapor pressure = 4 mm/Hg) and heptanal (HPH, vapor pressure = 3 mm/Hg). Contamination was not expected to be a major issue considering that the interval between odor deliveries was 4–5 minutes in our experiments.

### Delivering Binary Odor Mixtures

Two main considerations were taken into account in designing the flow pattern for binary mixtures of odors. First, we wished to maintain the concentration of individual component odors. Second, the olfactometer would be used in conjunction with recording of odor evoked response, some of which required tracheotomized animals to remove the influence of breathing. Since mouse olfactory neurons are sensitive to mechanical stimulation in addition to odors [31], it is important to maintain an overall constant air flow during odor mixture delivery to eliminate mechanical fluctuations. Therefore, the onset of odor delivery was accompanied by a corresponding drop in flow rate of the carrier stream to maintain the constant overall flow rate. The “off” command for odor delivery triggers corresponding increase in the carrier stream flow rate; and, because odor valves are controlled independently of each other, it is possible to deliver arbitrary overlapping sequences of odor mixtures.

We tested the ability of the olfactometers to deliver binary odor mixtures. Each olfactometer consisted of two odor banks, where one odor from each bank could be mixed to create sixteen different binary mixtures. Furthermore, each odor could be delivered at different flow rates, thus generating a large collection of possible concentration combinations. PID readout indicated that arbitrary odor mixtures could be generated by the olfactometer (Figure 3F), allowing versatile control of odor delivery.

### Automated Analysis of Odor Triggered Investigation

After investigating the ability of the olfactometers to generate and deliver binary mixtures, we tested the ease of using PROBES to examine odor triggered investigative behaviors. Specifically, we used PROBES to analyze odor detection in naïve animals that had not been previously exposed to the testing odors. We exploited the animals’ novelty seeking behavior to examine whether odor presentation induced odor port investigation, which we dubbed the habituation/dis-habituation assay.

**Figure 4:**
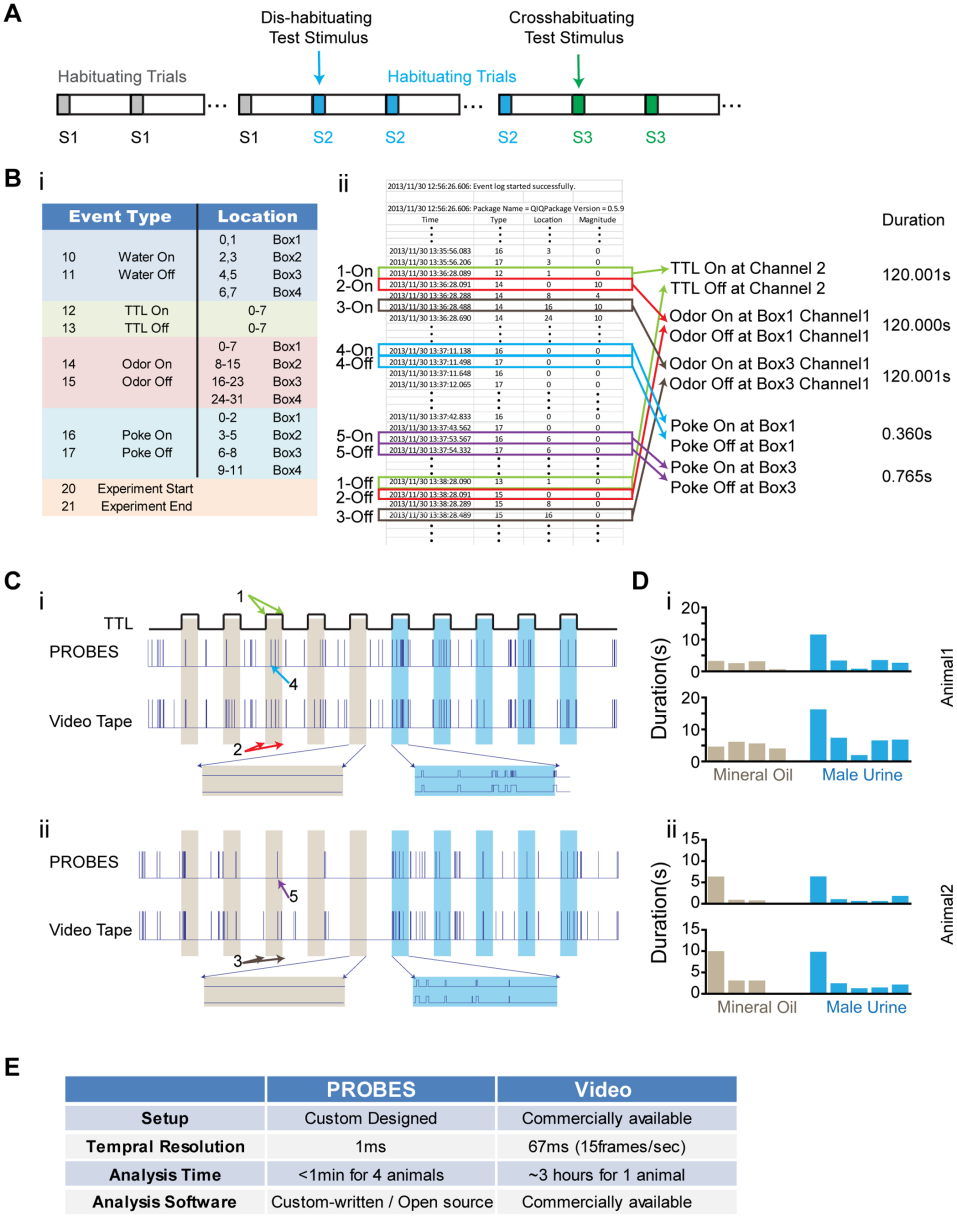
Behavior Assay Using PROBES. **A**) Schematic illustration of odor delivery sequence in dis-habituation and cross habituation assays. S1, S2, S3 indicate odor stimulus or air control. **B**) Event log from software package. (**i**) List of event types and corresponding device locations used in the log sheet. (**ii**) An example event log listing time, type, location and magnitude. Five pairs of events are labelled with different colors. The “on” and “off” events of each pair are indicated. Time elapsed between “off” and “on” indicates the duration of an event. **C**) Raster plots showing odor port investigation of two female animals (**i** and **ii**) in response to male urine registered by PROBES and with video recording. Enlarged views of activities during the last trial of mineral oil delivery and the first trial of male urine delivery are shown to illustrate the differences in investigation measurement between PROBES and video recording. TTL square pulses are marked to indicate odor delivery. Shaded columns indicate odor delivery of mineral oil (grey) and mouse urine (blue). Arrows 1–5 mark the five event pairs in **B**. **D**) Bar plots of the duration of odor investigation shown in **C**. The total time of odor port investigation in each trial was plotted. **E**) Comparison table for these two methods.

This assay required no prior training of the animal or previous exposure to any of the test odors. In a typical assay, when an animal was introduced to the test chamber, it began investigation of the odor port even when no odor was delivered. Repeated air delivery induced habituation in the animals manifested as a decrease in investigation of the odor port (Figure 4A). After the habituation period, an odor, considered as the dis-habituation stimulus, was delivered. The novelty of the presented odor resulted in a corresponding increase in port investigation. Repeated application of the odor stimulus caused habituation (Figure 4A). The sequence could be repeated, where continued presentation of a second odor following habituation to the first one allowed for examination of cross habituation between the two odors.

With PROBES, we were able to deliver odor stimulation and use IR-beam breaking events to register nose-poking activities. Each event, including TTL trigger, odor valve opening/closing and nose pokes, was time stamped and uniquely identified by its assigned location (digital input/output address) and event type (e.g., valve opening or closing) by the computer (Figure 4B). The event log was saved automatically and used for data analyses (Figure 4 B&C). The total amount of time of nose poke during odor delivery could be tallied to plot the level of investigation during different stimulus sessions (Figure 4D).

In order to evaluate the efficacy of PROBES, we compared the accuracy of event registration using our paradigm to classical video recording. The results obtained from PROBES analyses were qualitatively identical to those obtained from manual analysis of video recordings (Figure 4 C&D). Quantitatively, the duration of investigation was scored approximately 2×longer for video recordings than that obtained from PROBES. This was largely due to the difference in temporal resolution of the two methods. The temporal resolution for PROBES to register a single event was 1 millisecond and 67 ms for video recording (Figure 4E). In the example shown in Figure 4C, nose poke in and out of the odor port during odor investigation were registered as separate events by PROBES, but they could not be discerned in video inspection. For individual poking events, video inspection scores were slightly longer than those registered by PROBES. Additionally, during video analysis, loitering around the odor port might be subjectively scored as nose poking events by the experimenter. However, these discrepancies did not alter the overall results.

While the results were similar, the time required to analyze the behavior data differed significantly between the two methods (Figure 4E). Using PROBES, data from four animals was analyzed in a matter of minutes using a few custom-written MATLAB scripts. The same experimental data analyzed using video recordings required ∼12 hours of video transferring and manual scoring for all four animals.

### Cross Habituation Assay of Odor Discrimination

**Figure 5:**
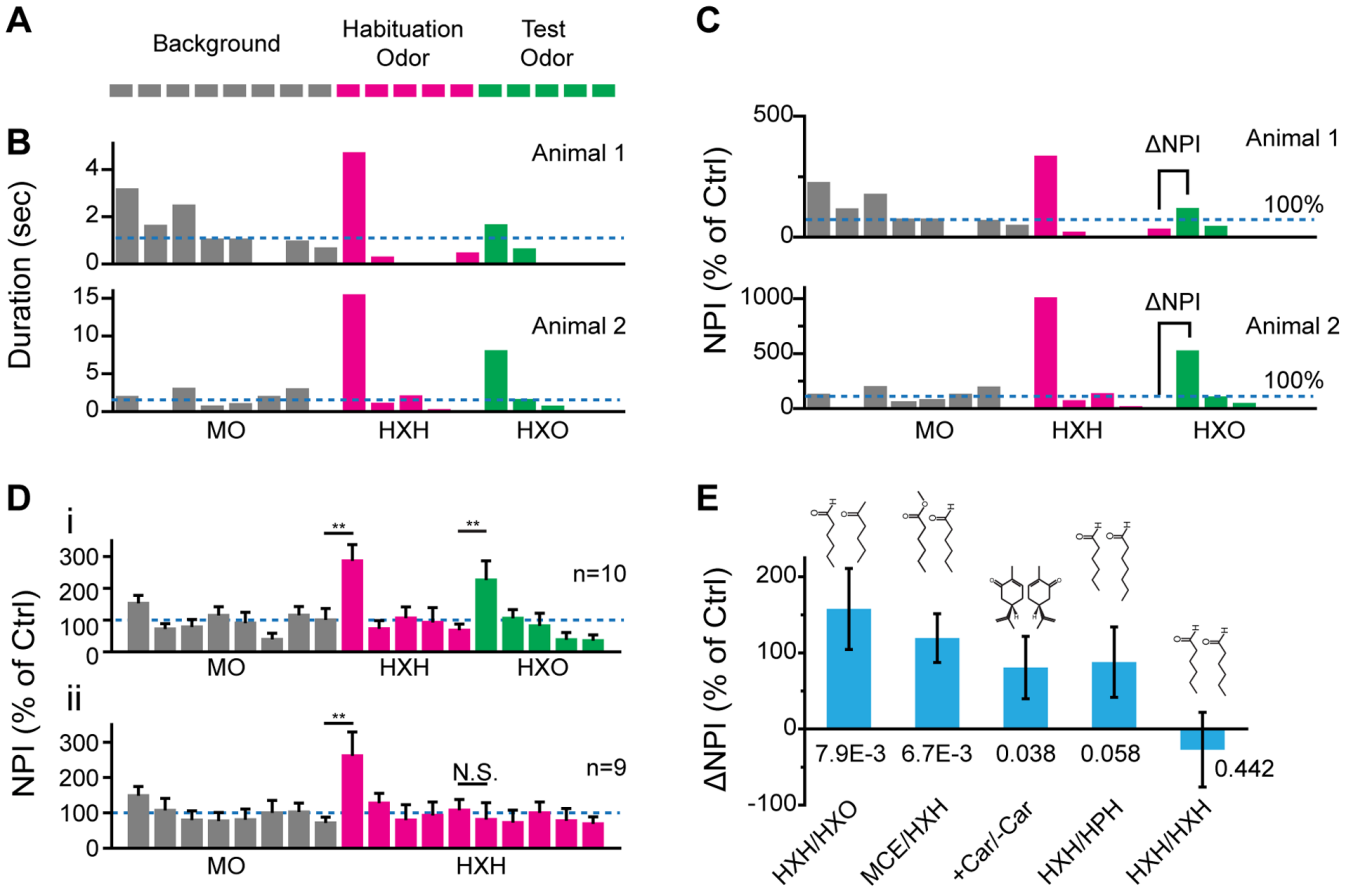
Cross Habituation Assay of Odor Discrimination. **A**) Schematic illustration of odor application sequence in a cross habituation assay. **B**) Bar plots of odor port investigation duration of two animals. Dashed lines indicate the average activity during eight sessions of mineral oil presentation. Odor dilution, 1∶1000. **C**) Bar plots of *NPI* from **B**. *ΔNPI* calculation is indicated. MO: mineral oil. **D**) Bar plots of average *NPI* in one cross habituation experiment. The number of animals tested is indicated; N.S., not significant. **: *p*<0.01. **E**) Bar plot of average *ΔNPI* for five odor pairs. Odor structures and pairwise *t-*test *p* values are shown. Error bars: S.E.M.

We further explored the use of PROBES for automated analysis of odor discrimination by adopting the cross habituation paradigm, which extended the habituation/dis-habituation paradigm by allowing habituation of the animal to repeated odor stimuli before application of a novel odor stimulus (Figure 5A). The response to the new odor was thought to indicate the perceptual differences between two odors [14], [15].

To reduce the influence of sensory adaptation, the cross habituation assay was designed to include a one minute odor stimulus at four minute intervals. In a previous study, a twenty second inter-stimuli interval was sufficient to allow the mouse olfactory system to respond robustly and consistently to the same odor with little sign of sensory adaptation [32].

We tested this paradigm using HXH and HXO (Figure 5). Following eight sessions of air habituation, the animals were exposed to five additional sessions of HXH. The first session gave rise to a significant increase in port investigation. Interest then dropped to background level and remained low through all subsequent sessions (Figure 5B&C). After habituation to HXH, a significant increase in investigation was observed during the first HXO presentation (Figure 5B&C). In contrast, continued presentation of HXH did not elicit increased investigation (Figure 5D).

During experimental sessions, individual animals exhibited different basal levels of activity, despite displaying similar habituation, dis-habituation and cross habituation behaviors (Figure 5B). To control for individual differences, we determined average port seeking during background air presentation ( as the basal activity. Investigation duration in each session was then normalized to obtain *normalized port investigation,* or *NPI* (Figure 5C):
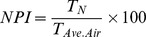



The difference between *NPI* values of two successive sessions (*ΔNPI*) reflected the level of cross habituation. For two consecutive sessions with the same odor stimulation, *ΔNPI* was approximately zero after the animals habituated to the odor (Figure 5D.ii). When a novel odor was presented after habituation, *ΔNPI* values provided a quantitative measure of cross habituation between the two odors (Figure 5C & 5D.i) and could be used as an indicator of perceptual difference between the two odors [14], [15] (Figure 5E). Thus the cross habituation assay allowed us to probe odor discrimination in mice that had never been exposed to the odors before.

### Two Choice Odor Discrimination Assay

Next, we tested the use of PROBES in studying trained olfactory behaviors. Specifically, we focused on odor discrimination in reinforced training assays. First, animals were tested in a two-choice assay using the triple-port chamber configuration (Figure 2B). During initial training, animals were presented a single odor associated with a fixed water port. Once the animals learned to seek the water port upon odor delivery, they were subject to two-choice training. Each training session contained a pseudorandom sequence of odor presentations (30 times AA, 30 times HPH). Water reward (5 μl) was given in contingent upon the animal’s correct port selection.

To measure performance, we defined success rate (*SR*) as:
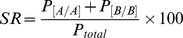
where *P_[A/A]_, P_[B/B]_* and *P_total_* were the number of pokes into A water port upon delivery of odor A, the number of pokes into B water port upon odor B and the total number of nose pokes into any water port, respectively.

**Figure 6:**
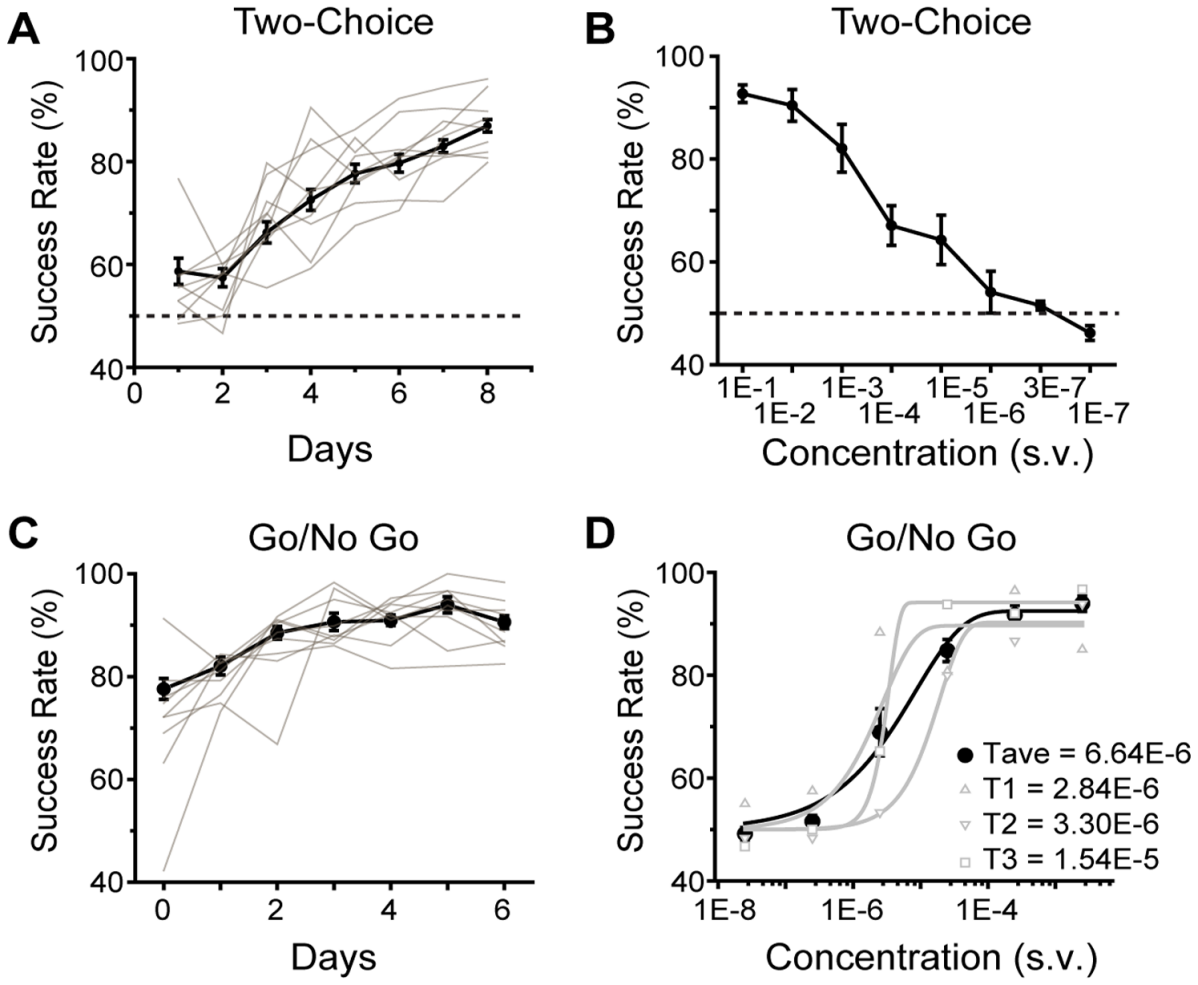
Reinforced Two-Choice Assay and Go/No Go Assay using PROBES. **A**) The learning curve in a two-choice assay to discriminate AA and HPH (1∶100 concentration, v/v) in 9 animals. Individual animals’ training curves are shown in grey lines. Error bars, S.E.M. **B**) Success rate (*SR*) in two-choice testing with decreasing odor concentration using the same set of animals as **A**. Chance level of discrimination is 50%, indicated by a dashed line. **C**) Training curve in a Go/No Go training using AA as reward odor (CS+, 1∶100, v/v) and MO (CS−). Individual animals training curves are shown in grey. **D**) Success rate in Go/No Go test with increasing odor concentration. The average *SR* from eight animals are indicated in black. The psychometric performances of three individual animals are plotted in grey. The results are fit with a Weibull psychometric function. Threshold values (T) calculated from the fitting are indicated in the figure.

Following seven days of training, mice could achieve >90% *SR* in discriminating AA from HPH (n = 9) (Figure 6A). We then examined the ability of the mice to perform odor discrimination task using decreasing concentrations of odors. In these tests, *SR* declined in correlation with odor concentration (Figure 6B). Remarkably, the animals were able to maintain *SR* above chance level at 10^−6^ dilutions. This is likely at the detection threshold of the odorants (see below).

### Go/No Go Assay

The Go/No Go test is another associative learning paradigm used to examine decision making and odor discrimination. In a Go/No Go test, an animal receives water reward when it correctly identifies the odor. Mis-identification of odors leads to no reward or punishment in the form of electrical foot-shock.

By altering the nose cone design (Figure 2C) and installing an electrical grid to deliver electric shocks triggered by the conditional TTL output from the olfactometer, we adapted the single port behavioral chamber for the Go/No Go assay. Mice with restricted water consumption were trained to receive a water reward by poking into the nose cone and licking the water spout. Next, crossing the first IR beam led to the delivery of either the conditioned stimulus (AA; CS+) or the aversive stimulus (HPH; CS−) in a pseudo-randomized sequence similar to two-choice training. CS+ odor delivery was associated with water reward, while CS− odor was coupled with a mild electric shock, when the mouse licked the water spout. No water was delivered upon CS− odor. Success rate (*SR*) was calculated as:
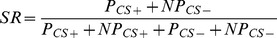
where *P_CS+_* and *NP_CS+_* were the number of licking and non-licking events for the CS+ odor, *P_CS_*_−_ and *NP_CS_*_−_ were number of the licking and non-licking events for the CS− odor respectively.

PROBES, when used to perform Go/No Go training sessions, was sufficient for odor/reward association. Animals were able to reach and maintain *SR* greater than 90% after five days of training (Figure 6C).

### Odor Detection Threshold

A reliable indicator of olfactory function is the ability to detect threshold amounts of odor. In human psychophysical tests, serial dilutions of odorants are presented to subjects, who verbally confirm whether they perceive the presence of an odor [33]. The “just noticeable difference” (JND) can also be used to determine detection threshold. Alternatively, human subjects are forced to determine whether or not they smell the presence of a particular odor when it is presented randomly at varying concentrations.

In animal research, the Go/No Go test is widely applied to examine detection threshold [7], [16], [21]. A major limitation of this assay is that it requires time consuming training. Animals that fail to complete the training must be excluded from further analysis. Neither does this method provide a means to examine odor detection by naïve animals. To address these issues, we evaluated the possibility of adopting the habituation/dis-habituation assay in conjunction with the Go/No Go test to determine odor detection threshold.

One group of animals were trained and tested using the Go/No Go test. After animals were trained to associate AA with water reward, we measured *SR* of licking events at different concentrations of AA (Figure 6D). The measured performance values could be fitted with the Weibull psychometric function [34], [35]. From the fitting, we derived the detection threshold of AA at 6.64×10^−6^ s.v. (Figure 6D). This result was consistent with that of a recent study [24].

**Figure 7:**
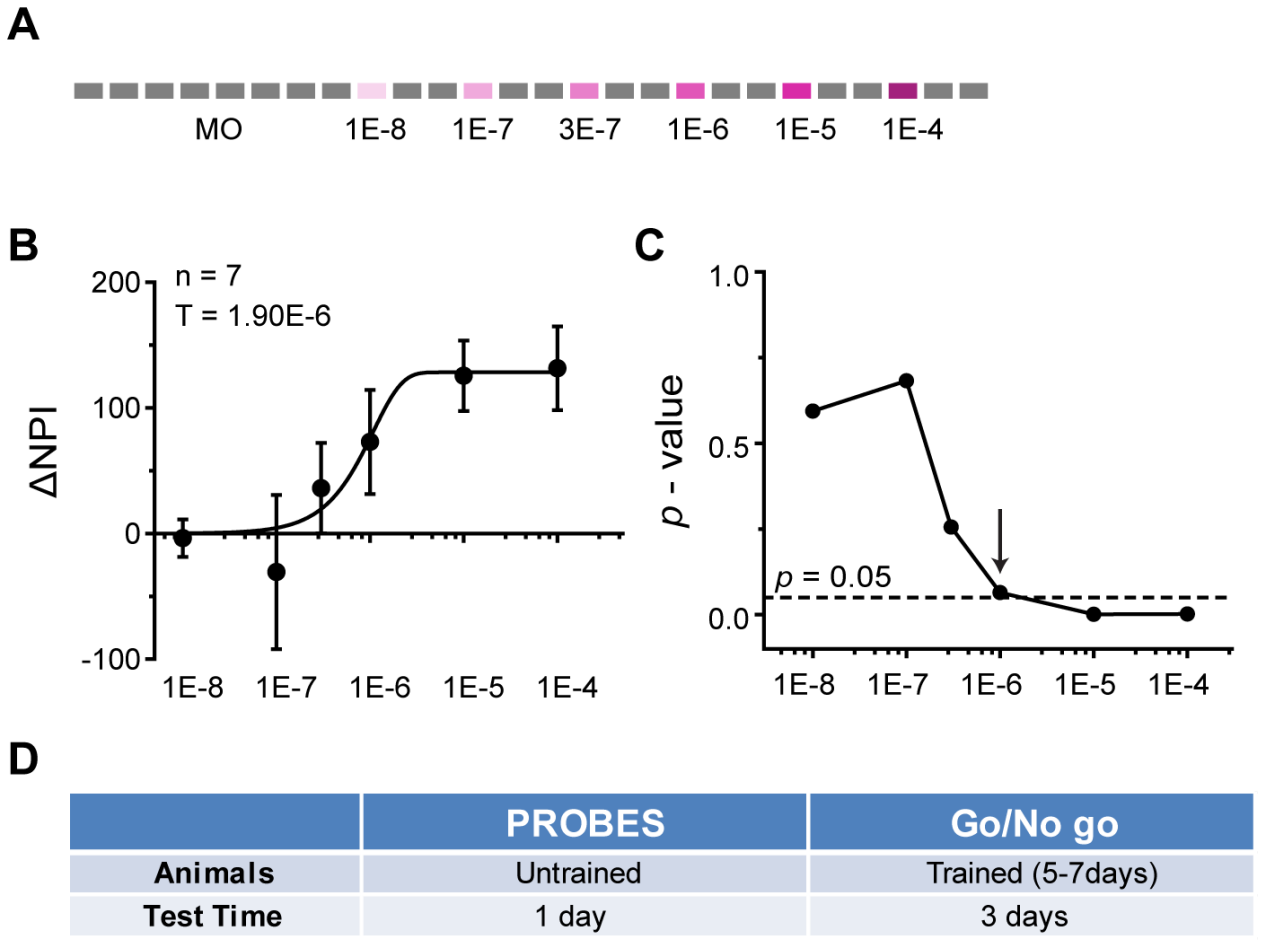
Habituation/Dis-habituation Assay for Measuring Odor Detection. **A**) Schematic illustration of odor application sequence in habituation/dis-habituation assay for threshold detection. Color intensity indicates increasing concentration of the test odor, amyl acetate. **B**) Average *ΔNPI* from 7 animals. The results are fitted with Weibull psychometric function. Threshold (T) values are indicated. **C**) Calculated *p* values from pairwise *t*-test of the *ΔNPI* are shown. Dotted line indicates *p* value at 0.05. Arrow indicates the odor concentration where the *p* value is approximate 0.05. **D**) Comparison table for PROBES and Go/No Go methods for threshold detection.

We used the one-chamber habituation/dis-habituation assay to examine detection threshold in a group of animals that had never been exposed to test odors. We habituated the animals with carrier air and delivered odors at increasing concentrations (Figure 7A). *ΔNPI* values, when plotted as a function of odor concentration (Figure 7B) and fit a psychometric curve with the Weibull psychometric function, indicated a detection threshold at 1.9×10^−6^ s.v.

The habituation/dis-habituation assay also provided information about the JND for mice, which could be analyzed by the statistical differences between the responses to an odor and its background. A significant increase in investigation of the odor port was expected to indicate detection (Figure 7C). Using this method, we found a significant increase in odor port investigation at 1×10^−6^ s.v., very close to the threshold determined by fitting with a psychometric curve (1.9×10^−6^ s.v., Figure 7B). Thus, experimentally determined detection thresholds in this assay were at a lower concentration than that obtained from the Go/No Go test. The difference likely resulted from the training involved in the Go/No Go assay (see Discussion).

These results demonstrated that odor detection threshold can be measured successfully and efficiently with PROBES using a simple habituation/dis-habituation assay rather than the more time consuming Go/No Go paradigm. With PROBES, we measured the response of up to twelve animals within one day. In contrast, the Go/No Go paradigm required seven days of training and three subsequent days of testing (Figure 7D).

### Testing Innate Odor Preference with PROBES

**Figure 8:**
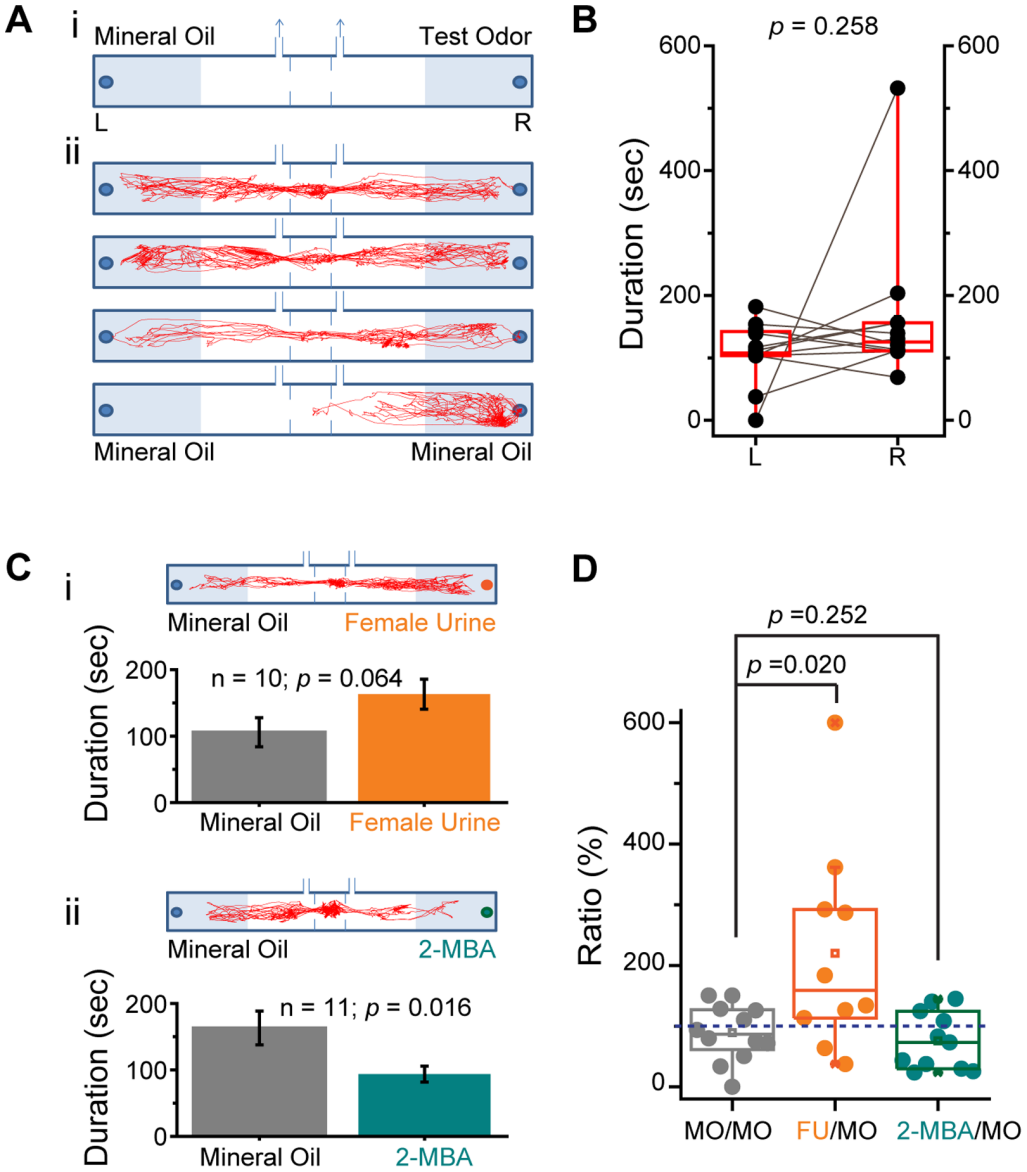
Three-chamber Test for Odor Preference. **A**) Schematic illustration of a classic 3-chamber design. The positions of odor vials are indicated. Shaded areas in each arm of the chamber indicate the odor zone for behavior analysis (**i**). Tracking traces of four animals in a control experiment with mineral oil vials in both chambers are shown (**ii**). **B**) Scatter plots of time spent in two zones in a control session with mineral oil. Each test session was 10 minutes. Twelve animals were examined. Pair wised *t*-test *p* value is indicated. **C**) Odor preference test for female urine (FU, **i**) and 2-MBA (**ii**). The upper panels show the tracking trace of one animal in the behavior chamber. Bar graphs indicate time spent in each zone. The number of animals (n) and *p* values of pairwise *t*-test are shown. **D**) Box plot of the ratio between test odor and MO. Dashed line indicates no difference between two zones. One-way ANOVA *p* values are indicated.

We explored the possibility of using PROBES to examine innate odor preference. Traditionally, odor attraction or aversion is measured using the two-chamber or three-chamber assays with video recording and manual analysis of animal activity (Figure 8A.i). With odor vials placed in separate chambers, the animals are allowed to freely explore each chamber and time difference spent in each chamber indicated odor preference.

We first performed classic three-chamber assays to examine the response to known aversive and attractive odors. In a control experiment, we placed mineral oil in both test chambers and video recorded the animals’ movement (Figure 8A). The time individual animals spent in shaded zones of each chamber was then quantified. Although on average spent similar amounts of time in each chamber, individual mice exhibited varying levels of spatial bias and chamber preference (Figure 8A&B). The ratio of time spent in the two chambers from different mice ranged from 0 to 1.57, with one mouse (out of twelve) residing solely in a single chamber during the test (Figure 8A&B).

After evaluating spatial bias, we next evaluated the animal’s response to a known aversive odor, 2-methylbutyric acid (2-MBA) [29], and mouse urine, a known attractive odor source. In the three-chamber assay, female urine appeared attractive to male mice (Figure 8C.i). However, simple paired *t*-test analysis between the two chambers indicated that the apparent attraction was not statistically significant (*p*>0.05). 2-MBA (10^−4^ s.v.) appeared to induce aversion, and paired *t*-test indicated a statistically significant difference (*p* = 0.016).

Considering the inherent variability in the two test chambers (Figure 8A&B), we performed ANOVA to determine the ratio between time spent in the two chambers. We found that the distribution in 2-MBA was not significant under more stringent statistical analysis (Figure 8D).

**Figure 9:**
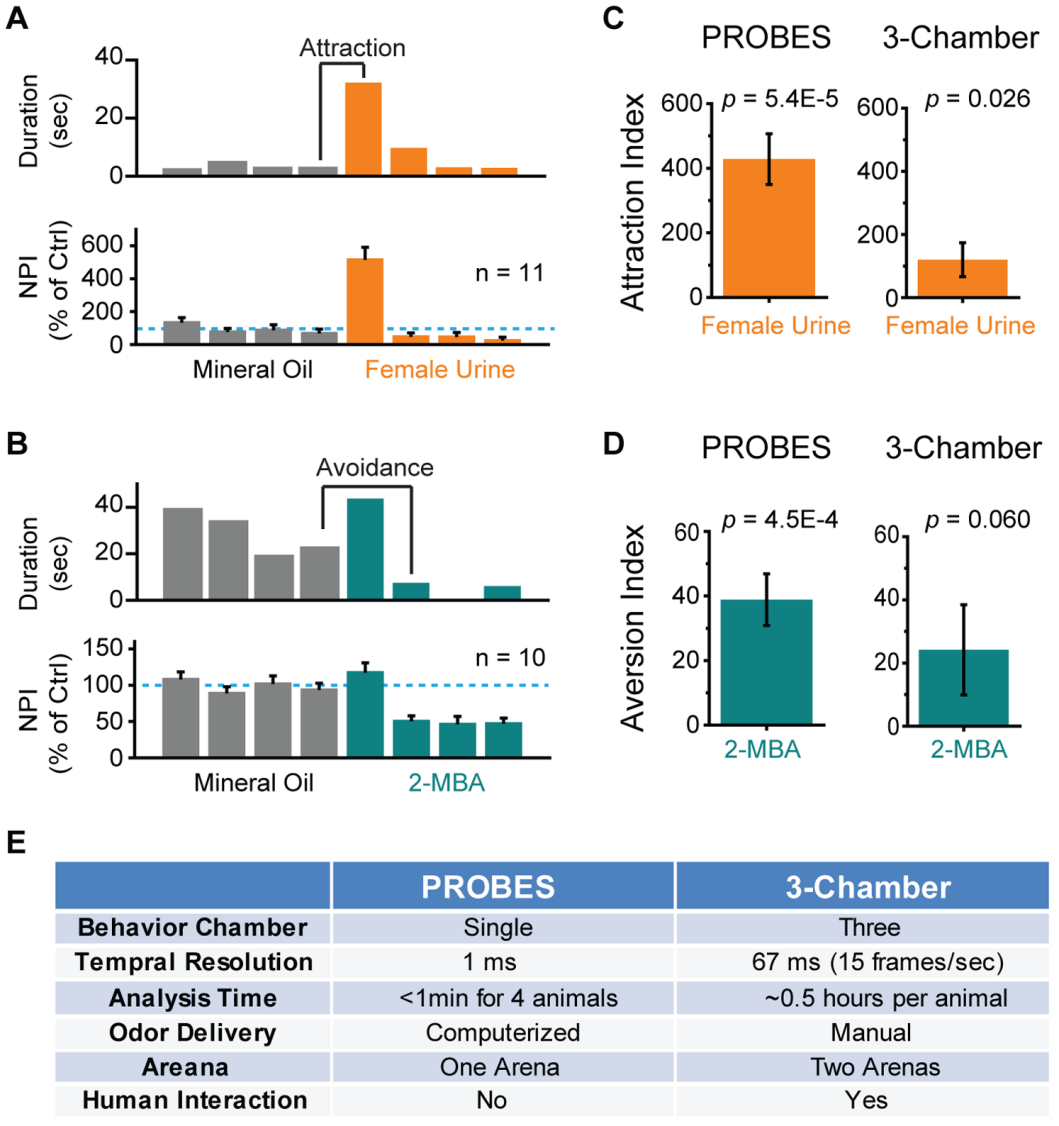
PROBES based habituation/Dis-habituation Analysis of Innate Odor Preference. **A&B**) Bar plots of odor port investigation by a single animal (upper panel) and the average of *NPI* value for FU (**A**, 10 animals) or 2-MBA (**B**, 11 animals). Dashed lines indicate 100% of average *NPI* to mineral oil. Calculation of attraction and aversion indexes is indicated. **C&D**) Comparison of attraction(**C**) and aversion (**D**) indexes obtained by PROBES assay and 3-chamber assays. The *p* values of the tests are indicated. **E**) Comparison table of PROBES and 3-chamber methods for innate preference assays.

We reasoned that the inherent variability in the three-chamber assay degraded the signal. We then explored the possibility of using a single chamber PROBES to examine the innate odor preference in a habituation/dis-habituation assay (Figure 9). Presentation of mouse urine elicited a significant increase in investigation (Figure 9A). In contrast, 2-MBA incited only a slight increase in investigation when first presented. Surprisingly, in subsequent 2-MBA sessions, the mice appeared to avoid approaching the odor port (Figure 9B). These observations indicated that *ΔNPI* value could be used to evaluate both attraction and aversion. Therefore, we calculated the *Attraction Index* by subtracting the *NPI* of last trial of mineral oil from the first trial of test odor (Figure 9A). Because aversion was better represented by the *ΔNPI* between the second odor presentation and final mineral oil control (Figure 9B), it was used as *Aversion Index*.

We also derived similar indices for the three-chamber assays by subtracting time spent in the control chamber from that in the test chamber to indicate attraction, and vice versa, to indicate aversion. The values were then normalized to the time spent in the control chamber. Comparison between PROBES and 3-chamber assay suggested that the PROBES-based habituation/dis-habituation assay yielded more robust readout with more statistically significant values (*p*<10^−4^ for mouse urine and *p*<10^−3^ for 2-MBA; Figure 9C&D). Moreover, PROBES-based assay and analysis took less time to perform (Figure 9E).

## Discussion

We have presented PROBES, an olfactometer-based behavioral apparatus for automated analysis of rodent behaviors. PROBES can be used efficiently and effectively to assess odor detection threshold, innate odor preference and odor discrimination. The automated nature of the system allows accurate measurement of odor-detection events free of subjective judgment, therefore eliminating variation among different experimenters. With PROBES, multiple animals can be assayed in parallel and the data can be processed within minutes. This makes it possible to increase the number of animals used in experimental groups to improve the statistical power of the studies.

One important application of PROBES is to examine olfactory behaviors in animals naïve to test odors. In our design, examinations of cross habituation, innate odor preference and odor detection threshold do not require time consuming odor-associated training. Since odor detection, perception and preference can be influenced by experience, using animals that are not previously trained can be critical in evaluating sensory functions. Indeed, we are able to use PROBES in cross habituation assays to examine odor discrimination. These assays complement the reinforced two-choice and Go/No Go assays in analyzing odor discrimination. The efficiency of the system permits the analyses of large numbers of odor pairs in a relatively short time span. Paradigms involving associative training, on the other hand, are time consuming and can become arduous when large numbers of animals and different odor pairs are used.

Moreover, the differences in *ΔNPI* values among different odor pairs may reflect quantitative differences in odor perception. In contrast, the two-choice assay, as well as Go/No Go test, is largely binary. The animals can be trained to discriminate different pairs of odors to similar levels of success rate.

PROBES offers a robust assay for innate odor preference. By comparing investigation of the same odor port triggered by different odorants, we reduce variability inherent in multi-chamber assays. However, using PROBES alone does not provide for behavioral parameters such as moving velocity, total activity and stimulus-induced freezing. Video recordings can be easily set up to obtain additional data for analysis.

Similarly, PROBES also offers an easy and accurate way to examine odor detection threshold. Using the habituation/dis-habituation assay, we can determine the odor detection threshold based on the difference between investigations of an odor and control air. This psychometric measurement differs from that of the Go/No Go test in that PROBES does not require extensive training. Therefore, we are able to examine odor detection in the naïve state of the animal. We observed a more sensitive measure of detection threshold using the habituation/dis-habituation assay than Go/No Go test, which may reflect the consequence of reinforced training. The behavioral readout – licking the water spout – is not only determined by odor detection, but also by a decision making process that evaluates the likelihood of reward. It is possible that animals choose not to act when odor is detected if the confidence level of obtaining a reward is low. They may perform an operant task at an odor concentration above threshold such that they have a higher probability to obtain reward and avoid punishment. It is also possible that reward-based training may enhance or inhibit sensitivity and skew the results. In habituation/dis-habituation test, animals are largely motivated by odor-driven curiosity to investigate the odor port. Odor detection at lower concentrations may be sufficient to trigger investigations without associated rewards. In the meantime, variation among individuals is corrected by normalizing the values to the control period. Under this condition, bias in decision making associated with stimulus valence is removed and the odor detection threshold measured under this naïve condition may reflect accurate odor sensitivity.

We must note that it is possible innately aversive odors may not trigger increased investigation. In this situation, we cannot use our method to determine detection threshold of these odors. For example, CO_2_ is an aversive stimulus to mice. The natural tendency for CO_2_ avoidance will likely hinder the use of habituation/dis-habituation assays to determine detection threshold. In this case, the Go/No Go assay will be a better choice [25].

In conclusion, PROBES is a versatile system that can be easily adapted to perform various olfactory tasks. By utilizing a single chamber design, we are able to assay odor-triggered behaviors without introducing biases associated with classical multi-chamber designs. The automated nature of the system not only eliminates human interaction with the test subjects, but also permits rapid sampling and analysis of data. These advantages together permit the efficient and effective examination of animal behaviors associated with the olfactory system.

## References

[1] Passe DH, Walker JC (1985) Odor psychophysics in vertebrates. Neurosci Biobehav Rev 9: 431–467. 3906450 10.1016/0149-7634(85)90021-1

[2] Slotnick BM, Kufera A, Silberberg AM (1991) Olfactory learning and odor memory in the rat. Physiol Behav 50: 555–561. 1801009 10.1016/0031-9384(91)90545-y

[3] Kepecs A, Uchida N, Zariwala HA, Mainen ZF (2008) Neural correlates, computation and behavioural impact of decision confidence. Nature 455: 227–231. 18690210 10.1038/nature07200

[4] Petrulis A, Eichenbaum H (2003) Olfactory memory. In: Doty RL, editor. Handbook of olfaction and gustation. 2nd ed. New York: Marcel Dekker. 409–438.

[5] Varley GC, Edwards RL (1953) An olfactometer for observing the behaviour of small animals. Nature 171: 789–790. 13054714 10.1038/172789a0

[6] Pfaffmann C (1958) An olfactometer for the rat. Science 128: 1007–1008. 13592287 10.1126/science.128.3330.1007

[7] Slotnick BM, Nigrosh BJ (1974) Olfactory stimulus control evaluated in a small animal olfactometer. Percept Mot Skills 39: 583–597. 4418626 10.2466/pms.1974.39.1.583

[8] Bodyak N, Slotnick B (1999) Performance of mice in an automated olfactometer: odor detection, discrimination and odor memory. Chem Senses 24: 637–645. 10587496 10.1093/chemse/24.6.637

[9] Lorig TS, Elmes DG, Zald DH, Pardo JV (1999) A computer-controlled olfactometer for fMRI and electrophysiological studies of olfaction. Behav Res Methods Instrum Comput 31: 370–375. 10495824 10.3758/bf03207734

[10] Darling FM, Slotnick BM (1994) Odor-cued taste avoidance: a simple and efficient method for assessing olfactory detection, discrimination and memory in the rat. Physiol Behav 55: 817–822. 8022899 10.1016/0031-9384(94)90065-5

[11] Duncan HJ, Beauchamp GK, Yamazaki K (1992) Assessing odor generalization in the rat: a sensitive technique. Physiol Behav 52: 617–620. 1409930 10.1016/0031-9384(92)90357-8

[12] Hubener F, Laska M (2001) A two-choice discrimination method to assess olfactory performance in pigtailed macaques, Macaca nemestrina. Physiol Behav 72: 511–519. 11282134 10.1016/s0031-9384(00)00447-9

[13] Hastings L, Miller M, Minnema D, Evans J, Radike M (1991) Effects of methyl bromide on the rat olfactory system. Chemical Senses 16: 43–55.

[14] Linster C, Smith BH (1999) Generalization between binary odor mixtures and their components in the rat. Physiol Behav 66: 701–707. 10386917 10.1016/s0031-9384(99)00007-4

[15] Cleland TA, Morse A, Yue EL, Linster C (2002) Behavioral models of odor similarity. Behav Neurosci 116: 222–231. 11996308 10.1037//0735-7044.116.2.222

[16] Mihalick SM, Langlois JC, Krienke JD, Dube WV (2000) An olfactory discrimination procedure for mice. J Exp Anal Behav 73: 305–318. 10866354 10.1901/jeab.2000.73-305 PMC1284779

[17] Moulton DG (1977) Minimum odorant concentrations detectable by the dog and their implications for olfactory receptor sensitivity. Chemical signals in vertebrates: Springer. 455–464.

[18] Rinberg D, Koulakov A, Gelperin A (2006) Speed-accuracy tradeoff in olfaction. Neuron 51: 351–358. 16880129 10.1016/j.neuron.2006.07.013

[19] Abraham NM, Spors H, Carleton A, Margrie TW, Kuner T, et al (2004) Maintaining accuracy at the expense of speed: stimulus similarity defines odor discrimination time in mice. Neuron 44: 865–876. 15572116 10.1016/j.neuron.2004.11.017

[20] Uchida N, Mainen ZF (2003) Speed and accuracy of olfactory discrimination in the rat. Nat Neurosci 6: 1224–1229. 14566341 10.1038/nn1142

[21] Walker JC, O’Connell RJ (1986) Computerized odor psychophysical testing in mice. Chemical Senses 11: 439–453.

[22] Wesson DW, Carey RM, Verhagen JV, Wachowiak M (2008) Rapid encoding and perception of novel odors in the rat. PLoS Biol 6: e82. 18399719 10.1371/journal.pbio.0060082 PMC2288628

[23] Barnes DC, Hofacer RD, Zaman AR, Rennaker RL, Wilson DA (2008) Olfactory perceptual stability and discrimination. Nat Neurosci 11: 1378–1380. 18978781 10.1038/nn.2217 PMC2682180

[24] Clevenger AC, Restrepo D (2006) Evaluation of the validity of a maximum likelihood adaptive staircase procedure for measurement of olfactory detection threshold in mice. Chem Senses 31: 9–26. 16306319 10.1093/chemse/bjj001

[25] Hu J, Zhong C, Ding C, Chi Q, Walz A, et al (2007) Detection of near-atmospheric concentrations of CO2 by an olfactory subsystem in the mouse. Science 317: 953–957. 17702944 10.1126/science.1144233

[26] Nigrosh BJ, Slotnick BM, Nevin JA (1975) Olfactory discrimination, reversal learning, and stimulus control in rats. J Comp Physiol Psychol 89: 285–294. 1176654 10.1037/h0076821

[27] Thiessen D, Lindzey G, Blum S, Wallace P (1971) Social interactions and scent marking in the Mongolian gerbil (< i> Meriones unguiculatus</i>). Animal Behaviour 19: 505–513. 10.1016/0003-3472(70)90065-5 5484039

[28] Doty RL (1975) Determination of odour preferences in rodents: A methodological review. Methods in Olfactory Research, Academic Press, New York: 395–406.

[29] Kobayakawa K, Kobayakawa R, Matsumoto H, Oka Y, Imai T, et al (2007) Innate versus learned odour processing in the mouse olfactory bulb. Nature 450: 503–508. 17989651 10.1038/nature06281

[30] Doty RL (2003) Methods for determining odor preferences in nonhuman mammals. In: Doty RL, editor. Handbook of olfaction and gustation. 2nd ed. New York: Marcel Dekker. 403–408.

[31] Grosmaitre X, Santarelli LC, Tan J, Luo M, Ma M (2007) Dual functions of mammalian olfactory sensory neurons as odor detectors and mechanical sensors. Nat Neurosci 10: 348–354. 17310245 10.1038/nn1856 PMC2227320

[32] Ma L, Qiu Q, Gradwohl S, Scott A, Yu EQ, et al (2012) Distributed representation of chemical features and tunotopic organization of glomeruli in the mouse olfactory bulb. Proc Natl Acad Sci U S A 109: 5481–5486. 22431605 10.1073/pnas.1117491109 PMC3325716

[33] Doty RL, Laing DG (2003) Psychophysical measurement of human olfactory function, including odorant mixture assessment. In: Doty RL, editor. Handbook of olfaction and gustation. 2nd ed. New York: Marcel Dekker. 203–228.

[34] Klein SA (2001) Measuring, estimating, and understanding the psychometric function: a commentary. Percept Psychophys 63: 1421–1455. 11800466 10.3758/bf03194552

[35] Wichmann FA, Hill NJ (2001) The psychometric function: I. Fitting, sampling, and goodness of fit. Percept Psychophys 63: 1293–1313. 11800458 10.3758/bf03194544

